# Sex-Dependent Effects of *Bmal1*-Deficiency on Mouse Cerebral Cortex Infarction in Response to Photothrombotic Stroke

**DOI:** 10.3390/ijms19103124

**Published:** 2018-10-11

**Authors:** Anne Lembach, Anna Stahr, Amira A. H. Ali, Marc Ingenwerth, Charlotte von Gall

**Affiliations:** 1 Institute of Anatomy II, Medical Faculty, Heinrich-Heine-University, Merowinger Platz 1A, 40225 Düsseldorf, Germany; annelembach@gmx.de (A.L.); stahranna@web.de (A.S.); amira.ali@med.uni-duesseldorf.de (A.A.H.A.); marc.ingenwerth@uk-essen.de (M.I.); 2 Institute for Pathology, University Hospital Essen, Hufelandstraße 55, 45147 Essen, Germany

**Keywords:** Bmal1, clock gene, photothrombotic stroke, cortical infarcts, reactive astrogliosis, activated microglia

## Abstract

Stroke is a leading cause of disability and death worldwide. There is increasing evidence that occurrence of ischemic stroke is affected by circadian system and sex. However, little is known about the effect of these factors on structural recovery after ischemic stroke. Therefore, we studied infarction in cerebral neocortex of male and female mice with deletion of the clock gene Bmal1 (*Bmal1*^−/−^) after focal ischemia induced by photothrombosis (PT). The infarct core size was significantly smaller 14 days (d) as compared to seven days after PT, consistent with structural recovery during the sub-acute phase. However, when sexes were analyzed separately 14 days after PT, infarct core was significantly larger in wild-type (*Bmal1*^+/+^) female as compared to male *Bmal1*^+/+^ mice, and in female *Bmal1*^+/+^, as compared to female *Bmal1*^−/−^ mice. Volumes of reactive astrogliosis and densely packed microglia closely mirrored the size of infarct core in respective groups. Estradiol levels were significantly higher in female *Bmal1*^−/−^ as compared to *Bmal1*^+/+^ mice. Our data suggests a sex-dependent effect and an interaction between sex and genotype on infarct size, the recruitment of astrocytes and microglia, and a relationship of these cells with structural recovery probably due to positive effects of estradiol during the subacute phase.

## 1. Introduction

Ischemic stroke is one of the major causes of disability and death worldwide. Risk factors include aging, high blood pressure, metabolic disturbances, as well as an “unhealthy” life style, such as smoking and obesity. While age-specific stroke rates are higher in men, women have relatively more stroke events because of their longer life expectancy [1]. In addition women are less likely to show excellent recovery [2]. There is a time-of-day dependent variation in the vulnerability to ischemia and in the occurrence of ischemic events [3,4], and shift work has been shown to be a risk factor for cardiovascular diseases in women [5]. Additionally, mice show a sex-dependent difference in stroke vulnerability [6] and a time-of-day dependent variation in neural damage after stroke [7]. Moreover, there is a sex-difference in the impact of shift work schedules on infarct volume and sensorimotor outcome in mice [8], suggesting an interaction between the two risk factors of female sex and chronodisruption. The circadian system drives rhythms in behavior, physiology, metabolism, and cognitive functions. The master circadian oscillator is located within the hypothalamic suprachiasmatic nucleus (SCN). The SCN orchestrates peripheral circadian oscillators in many tissues and organs. On the cellular level, the molecular clockwork is composed of interacting positive and negative transcriptional/translational feedback loops of clock genes [9,10]. This molecular clockwork drives rhythmic gene expression and thus rhythmic cell and organ function. The transcription factor BMAL1 (brain and muscle ARNT-like protein) is an essential component of the molecular clockwork. Mice with a targeted deletion of the *Bmal1* gene (*Bmal1*^−/−^) do not only show a complete loss of circadian rhythms, but also a severely reduced life span with various symptoms of premature aging as well as cognitive deficits [11,12,13,14,15,16]. We could show earlier that *Bmal1*-deficiency leads to an accelerated age-dependent decline in adult neurogenesis [15].

In this study, we analyzed the effects of *Bmal1*-deficiency and biological sex on mouse cerebral cortex infarction in response to focal cortical ischemia induced by photothrombosis (PT). The peri-infarct area surrounding the infarct core plays an important role in recovery and is an important therapeutic target during the sub-acute phase of ischemia. In mice, behavioral recovery is most prominent between six and 14 days (d) after PT [17]. Thus, we did not only analyze the infarct core but also volumes of reactive astrogliosis and densely packed microglia in the peri-infarct area seven days (d) and 14 days after PT.

Here, we demonstrate an interaction between circadian clock and sex on structural recovery in the peri-infarct area during the subacute phase of ischemia. Our findings point to an acceleration of structural recovery in the time interval between seven and 14 days after ischemic stroke by chronodisruption in female mice.

## 2. Results

### 2.1. Effect of Bmal1-Deficiency and Biological Sex on Infarct Core Volume

**Figure 1:**
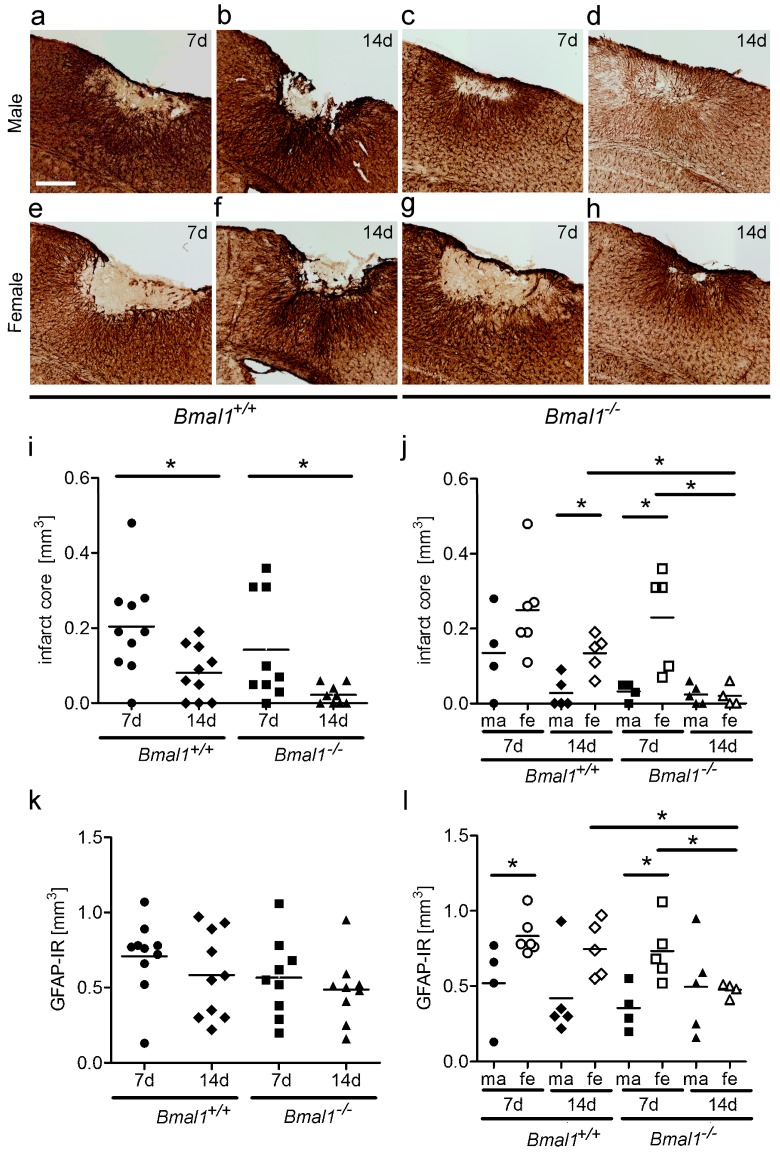
Effect of *Bmal1*-deficiency and sex on infarct core and astrocytes in the peri-infarct area. Infarct core and reactive astrogliosis in the peri-infarct area after focal cortical ischemia induced by photothrombosis (PT) were determined by GFAP-immunoreaction (IR). (**a**) *Bmal1*^+/+^ male 7 days (d) after PT; (**b**) *Bmal1*^+/+^ male 14 days after PT; (**c**) *Bmal1*^−/−^ male 7 days after PT; (**d**) *Bmal1*^−/−^ male 14 days after PT; (**e**) *Bmal1*^+/+^ female 7 days after PT; (**f**) *Bmal1*^+/+^ female 14 days after PT; (**g**) *Bmal1*^−/−^ female 7 days after PT; (**h**) *Bmal1*^−/−^ female 14 days after PT; (**i**) volume of infarct core in *Bmal1*^+/+^ and *Bmal1*^−/−^ mice of both sexes (*n* = 9–10 per genotype); and (**j**) volume of infarct core in *Bmal1*^+/+^ and *Bmal1*^−/−^ mice separated by sex (*n* = 4–5 per genotype and sex). (**k**) Volume of reactive astrogliosis in the peri-infarct area in *Bmal1*^+/+^ and *Bmal1*^−/−^ mice of both sexes (*n* = 9–10 per genotype); (**l**) Volume of reactive astrogliosis in the peri-infarct area in *Bmal1*^+/+^ and *Bmal1*^−/−^ mice separated by sex (*n* = 4–5 per genotype and sex). * *p* < 0.05, Mann Whitney-U Test. Scale bar = 300 µm.

The volume of infarct core (Figure 1a–h) was significantly smaller in both *Bmal1*^+/+^ mice and *Bmal1*^−/−^ mice 14 days (d), as compared to seven days after PT, when both sexes were taken together (Figure 1i). There was no significant difference in the infarct core volume between *Bmal1*^+/+^ mice and *Bmal1*^−/−^ mice seven days or 14 days after PT. In *Bmal1*^+/+^ mice, an infarct core volume of ≤0.1 mm^3^ could be observed in two from 10 (20%) mice seven days after PT and in six from 10 mice (60%) 14 days after PT (Figure 1i). In *Bmal1*^−/−^ mice, an infarct core volume of ≤ 0.1 mm^3^ could be observed in six from nine (66%) mice seven days after PT and in nine from nine mice (100%) 14 days after PT. However, when the sexes were analyzed separately, the infarct core volume was significantly smaller in female *Bmal1*^−/−^ mice 14 days compared to seven days after PT (Figure 1j). In *Bmal1*^+/+^ mice, infarct core volume was significantly larger in females as compared to males 14 days after PT (Figure 1j). All male *Bmal1*^+/+^ mice and none of the female *Bmal1*^+/+^ mice showed a regression of infarct core to a volume ≤0.1 mm^3^ 14 days after PT (Figure 1j). In *Bmal1*^−/−^ mice, a significantly larger infarct core volume was observed in females in comparison to males 7 days after PT (Figure 1j). However 14 days after PT, female *Bmal1*^−/−^ mice showed a significantly smaller infarct core volume as compared to female *Bmal1*^+/+^ mice (Figure 1j).

### 2.2. Effect of Bmal1-Deficiency and Biological Sex on Volumes of Reactive Astrogliosis and Densely Packed Microglia in the Peri-Infarct Area

The volume of reactive astrogliosis in the peri-infarct area (Figure 1a–h), as determined by the dense GFAP-immunoreactive area surrounding the infarct core, was not significantly different between *Bmal1*^+/+^ mice and *Bmal1*^−/−^ mice, when both sexes were analyzed together (Figure 1k). However, when the sexes were analyzed separately, the volume of reactive astrogliosis in peri-infarct area was only significantly smaller in female *Bmal1*^−/−^ mice 14 days compared to 7 days after PT (Figure 1l). In both *Bmal1*^+/+^ mice and *Bmal1*^−/−^ mice, the volume of reactive astrogliosis in the peri-infarct area was significantly larger in females as in males seven days after PT (Figure 1l). Consistent with the volume of infarct core, the volume of reactive astrogliosis in peri-infarct area was significantly smaller in female *Bmal1*^−/−^ mice as compared to female *Bmal1*^+/+^ mice 14 days after PT (Figure 1l).

**Figure 2:**
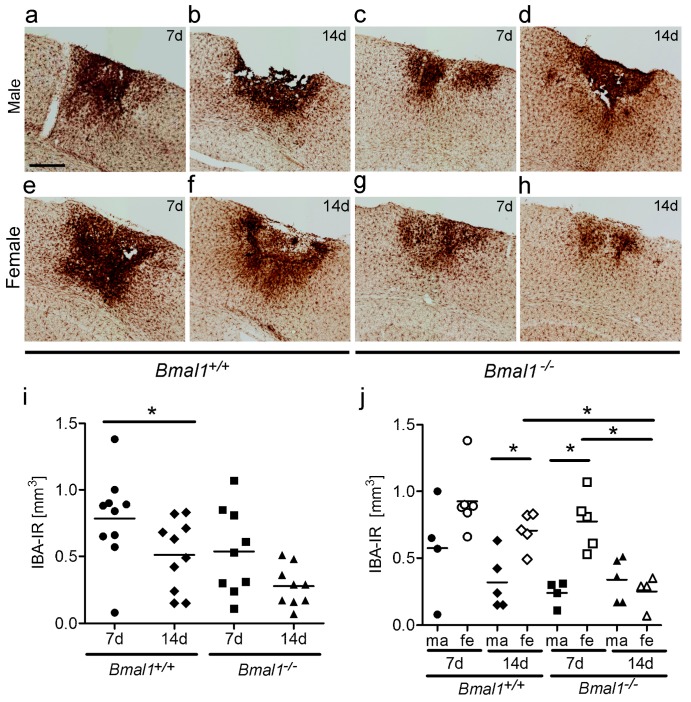
Effect of *Bmal1*-deficiency and sex on volume of densely packed microglia. Microglia after focal cortical ischemia induced by photothrombosis (PT) was determined by IBA-immunoreaction (IR). (**a**) *Bmal1*^+/+^ male 7 days after PT; (**b**) *Bmal1*^+/+^ male 14 days after PT; (**c**) *Bmal1*^−/−^ male 7 days after PT; (**d**) *Bmal1*^−/−^ male 14 days after PT; (**e**) *Bmal1*^+/+^ female 7 days after PT; (**f**) *Bmal1*^+/+^ female 14 days after PT; (**g**) *Bmal1*^−/−^ female 7 days after PT; (**h**) *Bmal1*^−/−^ female 14 days after PT; (**i**) volume of densely packed microglia in *Bmal1*^+/+^ and *Bmal1*^−/−^ mice of both sexes (*n* = 9–10 per genotype); and (**j**) volume of densely packed microglia in *Bmal1*^+/+^ and *Bmal1*^−/−^ mice separated by sex (*n* = 4–5 per genotype and sex). * *p* < 0.05, Mann Whitney-U Test. Scale bar = 300 µm.

The volume of densely packed microglia, as determined by the dense IBA-immunoreactive area (Figure 2a–h), was significantly smaller in *Bmal1*^+/+^ mice 14 days as compared to seven days after PT, when both sexes were taken together (Figure 2i). However, when the sexes were analysed separately, the volume of densely packed microglia was significantly smaller in female *Bmal1*^−/−^ mice 14 days after PT, as compared to seven days (Figure 2j). In *Bmal1*^+/+^ mice, the volume of densely packed microglia was significantly larger in females as compared to males 14 days after PT (Figure 2j). In *Bmal1*^−/−^ mice, the volume of densely packed microglia following ischemia was significantly larger in females as compared to males seven days after PT (Figure 2j). Consistent with the volumes of the infarct core and the reactive astrogliosis in the peri-infarct area, the volume of densely packed microglia was significantly smaller in female *Bmal1*^−/−^ mice as compared to female *Bmal1*^+/+^ mice 14 days after PT (Figure 2j).

### 2.3. Analyses of Newborn Cells in the Infarct Region

**Figure 3:**
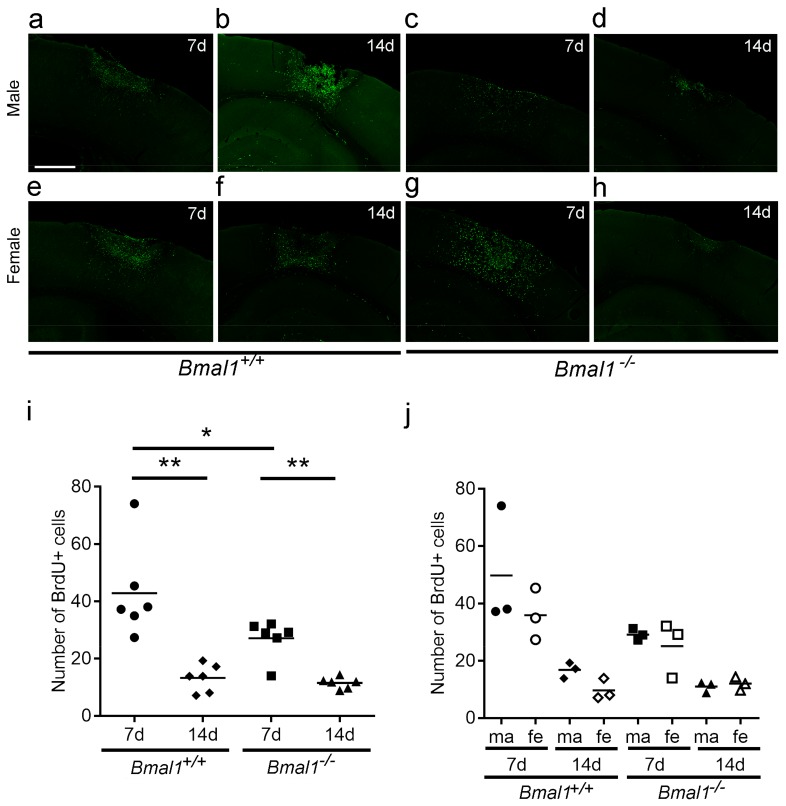
Effect of *Bmal1*-deficiency and sex on the number of newborn cells. Newborn cells in the peri-infarct area after focal cortical ischemia induced by photothrombosis (PT) were analysed by BrdU-immunoreaction. (**a**) *Bmal1*^+/+^ male 7 days after PT; (**b**) *Bmal1*^+/+^ male 14 days after PT; (**c**) *Bmal1*^−/−^ male 7 days after PT; (**d**) *Bmal1*^−/−^ male 14 days after PT; (**e**) *Bmal1*^+/+^ female 7 days after PT; (**f**) *Bmal1*^+/+^ female 14 days after PT; (**g**) *Bmal1*^−/−^ female 7 days after PT; (**h**) *Bmal1*^−/−^ female 14 days after PT; (**i**) number of BrdU-immunopositive (+) cells within the infarct region in *Bmal1*^+/+^ and *Bmal1*^−/−^ mice of both sexes (*n* = 6 per genotype); and (**j**) number of BrdU-immunopositive (+) cells within the peri-infarct area in *Bmal1*^+/+^ and *Bmal1*^−/−^ mice separated by sex (*n* = 3 per genotype and sex). * *p* < 0.05, ** *p* < 0.01, Mann Whitney-U Test. Scale bar = 300 µm.

For quantification of the total number of newborn cells in a defined region of the peri-infarct area, the number of BrdU^+^ cells was counted (Figure 3a–h). When both sexes were taken together, the number of newborn cells was significantly smaller in *Bmal1*^+/+^ and *Bmal1*^−/−^ mice 14 days compared to seven days after PT, and significantly smaller in *Bmal1*^−/−^ mice as compared to *Bmal1*^+/+^ mice 7 days after PT (Figure 3i). This reduction was, however, not observed when the animals were analyzed separately by gender (Figure 3j).

To further characterize the newborn cells, co-localization of BrdU with GFAP was analyzed as a measure for newborn astrocytes, and co-localization of BrdU with IBA was analyzed as a measure for newborn microglia.

**Figure 4:**
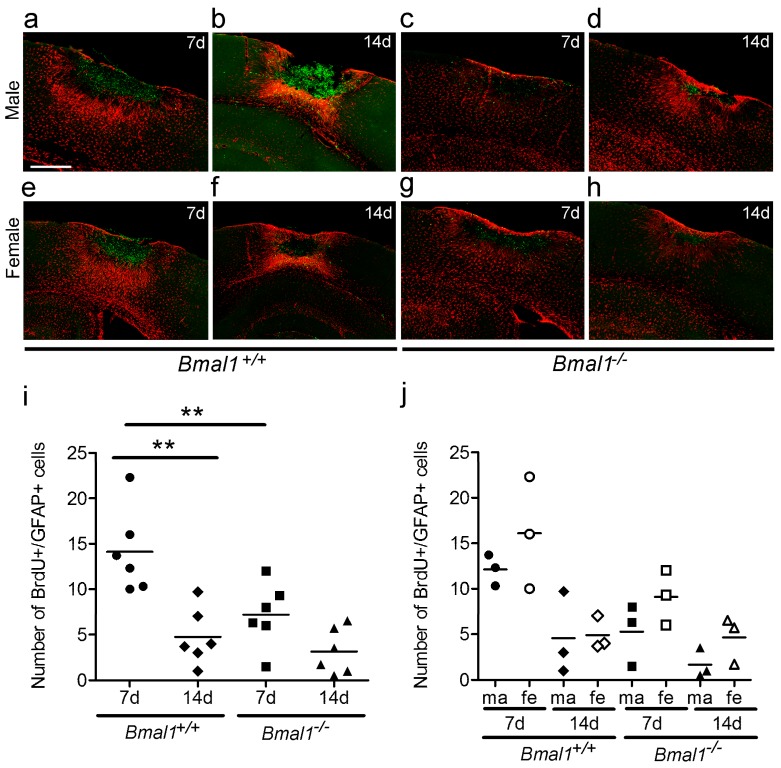
Effect of *Bmal1*-deficiency and sex on the number of newborn astrocytes. Newborn astrocytes in the peri-infarct area after focal cortical ischemia induced by photothrombosis (PT) were examined and analyzed by double-immunoreaction of BrdU and GFAP. (**a**) *Bmal1*^+/+^ male 7 days after PT; (**b**) *Bmal1*^+/+^ male 14 days after PT; (**c**) *Bmal1*^−/−^ male 7 days after PT; (**d**) *Bmal1*^−/−^ male 14 days after PT; (**e**) *Bmal1*^+/+^ female 7 days after PT; (**f**) *Bmal1*^+/+^ female 14 days after PT; (**g**) *Bmal1*^−/−^ female 7 days after PT; (**h**) *Bmal1*^−/−^ female 14 days after PT; (**i**) number of BrdU-/GFAP-immunopositive (+) cells within the peri-infarct area in *Bmal1*^+/+^ and *Bmal1*^−/−^ mice of both sexes (*n* = 6 per genotype); and (**j**) number of BrdU-/GFAP-immuno-positive (+) cells within the infarct region in *Bmal1*^+/+^ and *Bmal1*^−/−^ mice separated by sex (*n* = 3 per genotype and sex). ** *p* < 0.01, Mann Whitney-U Test. Scale bar = 300 µm.

The number of newborn astrocytes was significantly smaller in *Bmal1*^+/+^ mice 14 days as compared to seven days after PT and significantly smaller in *Bmal1*^−/−^ mice compared to *Bmal1*^+/+^ mice seven days after PT, when both sexes were taken together (Figure 4a–i). This closely reflects the results on the total number of newborn cells (Figure 3j). After the separation of sexes, no significant difference between male and female animals was observed (Figure 4j).

**Figure 5:**
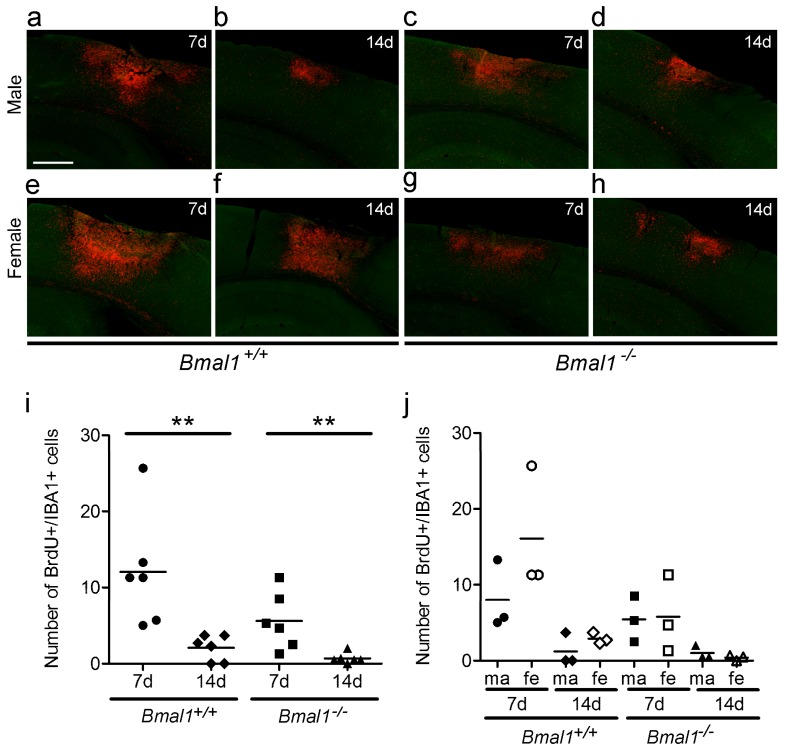
Effect of *Bmal1*-deficiency and sex on the number of newborn microglia cells. Newborn microglia cells in the peri-infarct area after focal cortical ischemia induced by photothrombosis (PT) were analysed by double-immunoreaction of BrdU and IBA. (**a**) *Bmal1*^+/+^ male 7 days after PT; (**b**) *Bmal1*^+/+^ male 14 days after PT; (**c**) *Bmal1*^−/−^ male 7 days after PT; (**d**) *Bmal1*^−/−^ male 14 days after PT; (**e**) *Bmal1*^+/+^ female 7 days after PT; (**f**) *Bmal1*^+/+^ female 14 days after PT; (**g**) *Bmal1*^−/−^ female 7 days after PT; (**h**) *Bmal1*^−/−^ female 14 days after PT; and (**i**) number of BrdU-/IBA-immunopositive (+) cells within the peri-infarct area in *Bmal1*^+/+^ and *Bmal1*^−/−^ mice of both sexes (*n* = 6 per genotype); (**j**) Number of BrdU-/IBA-immunopositive (+) cells within the infarct region in *Bmal1*^+/+^ and *Bmal1*^−/−^ mice separated by sex (*n* = 3 per genotype and sex). ** *p* < 0.01, Mann Whitney-U Test. Scale bar = 300 µm.

The number of newborn microglia was significantly smaller in both genotypes 14 days when compared to seven days after PT (Figure 5a–h), when both sexes were taken together (Figure 5i). After separation of sexes, there was no significant difference among the 8 groups (Figure 5j).

Double staining for BrdU and the neuronal marker NeuN confirmed the absence of newly proliferated mature neurons (data not shown), which is consistent with several studies following focal ischemia in rats and mice [17,18].

### 2.4. Analysis of GnRH-Immunoreaction and Estradiol Levels

**Figure 6:**
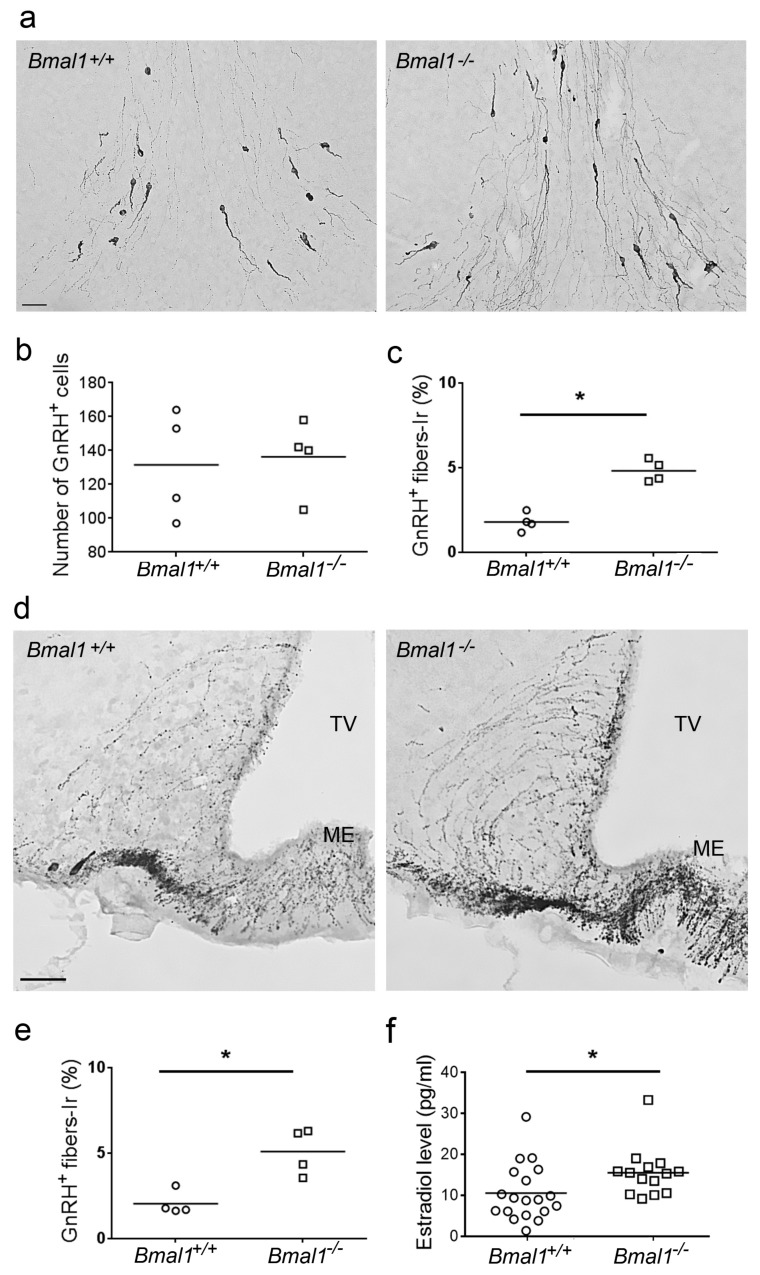
Effect of *Bmal1*-deficiency on gonadotropin releasing hormone and estrogen in female mice. Gonadotropin releasing hormone (GnRH) neurons and fibers were identified by GnRH-immunoreaction (*n* = 4 per genotype), estrogen plasma levels were determined by ELISA (*n* = 14–19 per genotype). (**a**) GnRH-immunoreaction in the preoptic area of *Bmal1*^+/+^ female and *Bmal1*^−/−^ female mice. (**b**) Number of GnRH-immunopositive (+) cells in the preoptic area of *Bmal1*^+/+^ female and *Bmal1*^−/−^ female mice. (**c**) Percentage of GnRH-immunopositive (+) fibers in the preoptic area of *Bmal1*^+/+^ female and *Bmal1*^−/−^ female mice. (**d**) GnRH-immunoreaction in the median eminence of *Bmal1*^+/+^ female and *Bmal1*^−/−^ female mice. (**e**) Percentage of GnRH-immunopositive (+) fibers in the median eminence of *Bmal1*^+/+^ female and Bmal1^−/−^ female mice. (**f**) Levels of plasma estradiol in *Bmal1*^+/+^ female and *Bmal1*^−/−^ female mice. Third ventricle (TV), Median eminence (ME), * *p* < 0.05, Mann Whitney-U Test. Scale bars = 50 µm.

As only in females, differences in the volume of infarct core, glial scar, microglial activation, and number of newborn cells in the per-infarct area between *Bmal1*^+/+^ and *Bmal1*^−/−^ mice were observed, we tested GnRH-immunoreaction and plasma estradiol levels. While the number of GnRH-immunoreactive cells did not differ between female *Bmal1*^+/+^ and *Bmal1*^−/−^ mice (Figure 6a,b) the amount of GnRH-immunoreactive fibers was significantly higher in female *Bmal1*^−/−^ compared to *Bmal1*^+/+^ mice in both the preoptic area (Figure 6a,c) and the medio-basal hypothalamus (Figure 6d,e). Moreover, the plasma estradiol levels were significantly higher in female *Bmal1^−/−^* compared to female *Bmal1*^+/+^ mice (Figure 6f).

## 3. Discussion

In this study we analyzed the effect of Bmal1-deficiency, as a model for circadian rhythms disruption, and biological sex on the volume of infarct core, reactive astrogliosis, and densely packed microglia in peri-infarct area during the subacute stage after focal ischemic stroke induced by photothrombosis (PT).

In both *Bmal1*^+/+^ and *Bmal1*^−/−^ mice, volume of infarct core was significantly reduced 14 days (d) compared to seven days after PT. This is consistent with a time-dependent decrease in the infarct size during the late acute stage (3–6 d) and the sub-acute stage (7–31 d) in rodents such as rats [19,20,21] or mice [17,22] after focal ischemic stroke induced by PT. A similar time-dependent regression in infarct size can be observed in human patients after ischemic stroke by means of serial follow-up diffusion-weighted magnetic resonance imaging [23,24,25]. When both sexes were taken together, there was no significant difference in the infarct core volume between *Bmal1*^+/+^ and *Bmal1*^−/−^ mice 7 days or 14 days after PT. Recent findings showed a pro-thrombotic phenotype in *Bmal1*-deficient mice associated with an increased platelet aggregation [26] and a reduced stress response [27]. While these vascular factors could be considered to play a modulatory role in stroke outcome in Bmal1-deficient mice, *Bmal1*-deficiency per se does, according to our results, not affect regression of the infarct core.

However, when the sexes were analyzed separately, we found significant differences between males and females as well as between females of both genotypes even within this small sample size. This suggests effects of sex and genotype on infarct core volume. Surprisingly, 14 days after PT, the volume of infarct core was significantly larger in female *Bmal1*^+/+^ mice, as compared to *Bmal1*^−/−^ mice. This was unexpected, as acutely after stroke (one and three days), the infarct core is larger in male mice, as compared to female mice [28]. This indicates that biological sex does not only affect the vulnerability but also the dynamic of recovery after stroke.

Importantly, Earnest et al. showed an interaction between circadian rhythm disruption and biological sex to modulate pathological effects of stroke [8]. They showed, that in rats kept under a shift work like light/dark 12h:12h (LD) cycle, males but not females showed a significant increase in mortality after middle cerebral artery occlusion (MCAo). Five days after MCAo, the surviving female rats kept under shift-work like conditions showed a significantly higher infarct volume and sensorimotor deficits relative to female rats kept under non-shift work like conditions [8]. Furthermore, ambient light induced an evident sex-dependent response of body temperature and blood pressure only in females, indicating a crucial sex-related difference in the circadian regulation of cardiovascular functions [29]. In our study, there was no significant difference in infarct core volume between female *Bmal1*^+/+^ and *Bmal1*^−/−^ mice sacrificed seven days after PT. However, volumes of infarct core, astrocytes and densely packed microglia in the peri-infarct area were significantly smaller in female *Bmal1*^−/−^ mice as compared to female *Bmal1*^+/+^ sacrificed 14 days after PT. This shows that in females, circadian rhythm disruption as a consequence of shift work-like schedule has a negative effect of infarct progression during the acute stage [30], whereas circadian rhythm disruption as a consequence of *Bmal1*-deficieny has a positive effect on infarct progression during the sub-acute stage (this study). Female rats under shift-work like conditions showed persistent estrus associated with increased estradiol levels and decreased IGF-1 levels [8]. This is consistent with changes in morphology of GnRH-neurons and increased estradiol levels observed in our study as well as disturbed estrus cycle in *Bmal1*-deficient mice [31,32]. Furthermore, previous findings point to an interaction between the *Bmal1* gene and the estrogen receptor 1 gene (ESR1), encoding the estrogen receptor (ER), which has shown to mediate the neuroprotective functions of estrogen [33]. There is ample evidence for the neuroprotective effect of estrogen in ischemia [28,34,35,36]. There are, however, other factors beyond estrogen that have been shown to modulate sex differences in circadian timing systems [37,38] and could consequently have influenced the findings of this work. These factors should, consequently, also be taken into consideration for further research.

Following ischemic or traumatic brain injury, astrocytes exhibit multiple changes, and are known to become reactive [39,40,41]. In this reactive state, the so-called reactive astrogliosis, astrocytes are characterized by an excessive expression of GFAP and depending on the extent of damage eventually form a glial scar surrounding the central ischemic core [20,42,43]. Moreover, focal ischemic stroke induces a context-specific immune reaction. Microglia are the resident immune cells of the CNS and undergo activation after pathological events. There is evidence that microglia provide essential protective functions after focal ischemia [44,45]. Therefore, we examined the volume of reactive astrogliosis and microglia surrounding the infarct core. When both sexes were analyzed together, we did not find a significant difference in the volume of the astrocytes in peri-infarct area between seven days and 14 days after PT in both genotypes. This is consistent with the dynamic of GFAP-immunopositive cells in the vicinity of the infarct showing a high increase during the acute phase (two–four days after PT) and no significant change during the sub-acute phase (six–14 days after PT) [17]. Thus, in the sub-acute phase the time-dependent decline in infarct core is not strongly correlated with a time-dependent decline in the volume of reactive astrogliosis in the peri-infarct area. However, when the sexes were analyzed separately, we found a significantly larger volume of reactive astrogliosis in peri-infarct area in females as compared to males seven days after PT in both genotypes. This suggests a sex-dependent effect on the formation of the glial scar during the sub-acute phase. The volume of densely packed microglia was significantly smaller 14 days, as compared to seven days after PT in *Bmal1*^+/+^ mice. This is consistent with a decrease in the infarct core volume. Moreover, when the sexes were analyzed separately, we found the same differences between sexes and genotypes in the volume of densely packed microglia as in the volume of the infarct core. This suggests, that during the sub-acute stage the effects of sex and genotype on infarct volume and densely packed microglia activation are closely related. Following ischemic injury, astrocytes and microglia have been shown to undergo a variety of changes, such as hypertrophy and proliferation [43]. The total number of proliferating cells, proliferating astrocytes, and proliferating microglia in the peri-infarct area was smaller seven days compared to 14 days after PT in *Bmal1*^+/+^ mice. Thus, there is a time-dependent decrease of cell, astrocyte and microglia-proliferation during the sub-acute phase. However, there was no sex-dependent difference in the total number of proliferating cells, proliferating astrocytes or proliferating microglia. Therefore, the sex-dependent differences in infarct core volume are presumably not a consequence of newly born astrocytes or microglia, but rather due to the activation of resident astrocytes and microglia of the peri-infarct area.

In summary, our data show a sex-dependent difference and a sex-dependent effect of Bmal1-deficiency on infarct core volume during the sub-acute phase after focal cortical ischemia. These effects do not seem to be dependent on proliferation, but activation or recruitment of astrocytes and microglia in the peri-infarct area. In conclusion, our results support the hypothesis of an interaction between biological sex and chronodisruption on structural recovery after stroke.

## 4. Material and Methods

### 4.1. Experimental Animals

All animal procedures were performed in accordance with animal welfare regulations and experimental protocols were approved by the local government authority LANUV (North Rhine-Westphalia State Agency for Nature, Environment and Consumer Protection; Germany; Reference numbers: 84-02.04.2012.A380, initial approval date: 26 June 2013, approval date of amendments: 25 February 2014, 7 October 2014; 84-02.04.2014.A314, approval date: 9 December 2014). Heterozygous mice with a targeted deletion of *Bmal1* (*Bmal1*^+/−^) were obtained from Jackson Laboratories (B6.129-*Arntl^tm1Bra^*/J) and kept for breeding at the local animal facility of University of Düsseldorf to obtain *Bmal1*^−/−^ and *Bmal1*^+/+^ littermates. PCR was used to determine the genotype [11]. Mice were housed in standard cages, with 12 h light/12h darkness with free access to food and water. Male and female *Bmal1*^+/+^ and *Bmal1*^−/−^ littermates (8–12 weeks old) were used. At this age, the *Bmal1*^−/−^ mice do not show global astrogliosis [13]. This was confirmed by morphological analysis of GFAP-immunoreactive astrocytes contralateral to the focal cortical ischemia.

### 4.2. Induction of Cortical Stroke by Photothrombosis

For induction of focal cortical ischemia, photothrombosis (PT) was applied. Adult *Bmal1*^+/+^ and *Bmal1*^−/−^ mice were anesthetized by 2–2.5% isoflurane in mixture of nitrous oxide and oxygen. After stereotactic fixation of the head (Stereotakte, Digital Stereotaxic Instrument, 51730D, Stoelting Co., Wood Dale, IL, USA), the scalp was shaved and opened. The cold light source (1.5 mm diameter, 2500 Lux, KL1500 LCD, Schott, Mainz, Germany) was placed 2.0 mm dorsal and 2.5 mm lateral from bregma. After the injection of the photosensitive dye Rose Bengal (43 mg/kg body weight dissolved in 0.9% saline, Sigma Aldrich, St. Louis, MO, USA) into the tail vein, the cold light source was applied for 15 min. The activation of the photosensitive Rose Bengal leads to the release of free oxygen radicals in the area of light exposure, which results in a local endothelial damage with subsequent aggregation of platelets. Consequently, the local disruption of blood supply leads to an irreversible damage on the tissue [22,46]. For analgesic treatment, carprofen (Rimadyl^®^, 4 mg/kg body weight dissolved in 0.9% saline) was injected subcutaneously during surgery as well as for two consecutive days after surgery. The mice were sacrificed 7 days or 14 days after PT, as at 7 days after PT the astrocytes in the region surrounding the infarct core are densely packed and start to form a glial scar [17,19,22] and 14 days after PT, the activation of microglia in the lesion is fully developed [20]. In human patients, the time frame from 7 days to 6 month after stroke is considered as the subacute stage [30].

### 4.3. BrdU Assay for Proliferating Cells

For quantification of newly born cells after induction of focal cortical infarct by PT, the proliferation marker 5-Brom-2′-desoxyuridin (BrdU (Roche, Basel, Switzerland) 100 mg/kg body weight dissolved in 0.9% saline) was injected intraperitoneally on three consecutive days. One group of mice was injected with BrdU from the third to the sixth post-ischemic day and the mice were sacrificed one day after the last injection (7 days). The second group was injected with BrdU from the eighth to thirteenth post-ischemic day and sacrificed on day 14 after PT (14 d).

### 4.4. Perfusion and Tissue Preparation

Mice were deeply anaesthetized using ketamine: xylazine (100 mg: 10 mg/kg body weight) and transcardially perfused with 0.9% NaCl followed by 4% paraformaldehyde in phosphate buffered saline (PBS). Brains were removed and post fixed in 4% paraformaldehyde (in PBS) for 24 h and cryoprotected in 20% sucrose in PBS for another 24 h. Neocortex with infarct area was sectioned coronally (30 µm thickness) by using a cryomicrotome (Reichert-Jung, New York, NY, USA) in 12 parallel series. Each parallel series was used for immunohistochemistry or immunofluorescence with a different antibody.

### 4.5. Immunohistochemistry and Analyses of Volumes of Infarct Core, Reactive Astrogliosis in Peri-Infarct Area and Densely Packed Microglia

Free-floating sections were washed in washing butter (0.2% Triton-X 100 in PBS) and then incubated with 0.24% H_2_O_2_ in PBS for 30 min at room temperature. After further washing, sections were incubated with the primary antibody against the astrocytic marker GFAP (1:1500, Dako Agilent Technologies, Santa Clara, CA, USA, Z0334) or the microglial marker IBA1 (1:500, Wako Industries, Osaka, Japan, 019-19741) overnight at 4 °C, followed by goat anti-rabbit IgG (1:500, Vector Laboratories, Burlingame, CA, USA, ZA0520) for 2 h at room temperature. After further washing, sections were incubated with Vectastain Elite ABC Kit (Vector Laboratories) for 1 h followed by incubation in 3.3’-diaminobenzidine (Sigma-Aldrich) in the presence of 0.3% hydrogen peroxide for 10 min. Sections were washed with PBS/0.2% Triton-X 100, mounted on microscope slides. Slides were air-dried and cover-slipped with Entellan (Merck Millipore, Danvers, MA, USA).

**Figure A1:**
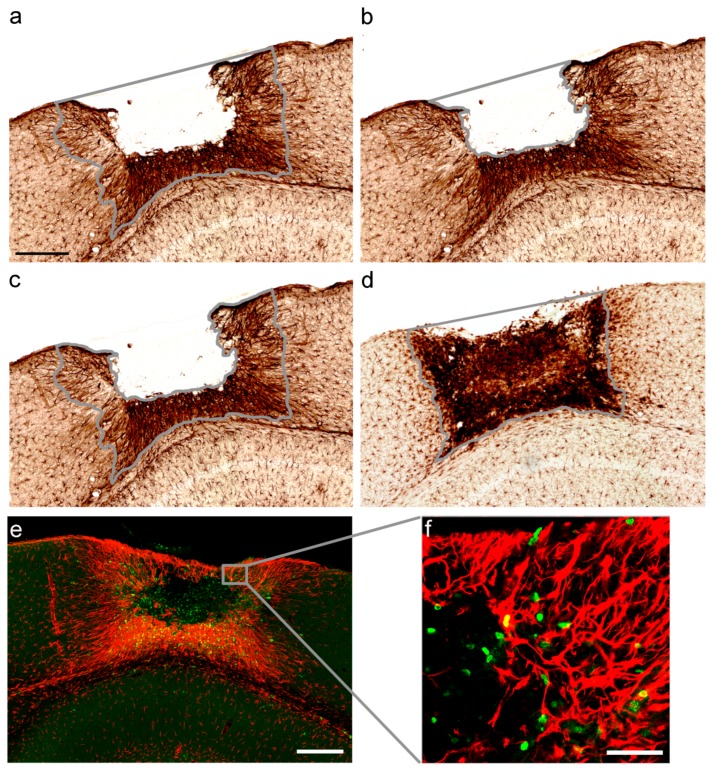
Definition of infarct region, infarct core, peri-infarct area, area of densely packed microglia and part of the peri-infarct area for quantification of newborn cells. (**a**) Infarct region including the infarct core and peri-infarct area; (**b**) infarct core; (**c**) reactive astrogliosis in peri-infarct area was determined by GFAP-immunoreaction and indicated by freehand drawing in a representative section. (**d**) Area of densely packed microglia was determined by IBA1-immunoreaction and indicated by freehand drawing in a representative section. Scale bar = 300 µm. (**e**) Part of the peri-infarct area for quantification of newborn cells is indicated by the box in a representative section. Scale bar = 300µm, (**f**) Boxed region from (**e**) at higher magnification. Scale bars = 50 µm.

Infarct core corresponds to the central ischemic core and peri-infarct area was defined as the area of reactive astrogliosis and microglia surrounding the infarct core [47,48]. Volumes of infarct core, reactive astrogliosis and densely packed microglia in the peri-infarct area were analyzed 7 days and 14 days after PT in male and female *Bmal1*^+/+^ mice (7d ♂ *Bmal1^+/+^ n* = 4; 7d ♀ *Bmal1*^+/+^ *n* = 6; 14d ♂ *Bmal1*^+/+^ *n* = 5; 14d ♀ *Bmal1*^+/+^ *n* = 5) and *Bmal1*^−/−^ mice (7d ♂ *Bmal1*^−/−^ *n* = 4; 7d ♀ *Bmal1*^−/−^ *n* = 6; 14d ♂ *Bmal1*^−/−^ *n* = 5; 14d ♀ *Bmal1*^−/−^ *n* = 4). Image analysis was performed by an observer blind to the experimental condition. Volume of infarct core and reactive astrogliosis in peri-infarct area were determined by GFAP-immunoreaction (IR) (Figure A1a–c in Appendix A). Volume of densely packed microglia was determined by IBA1-IR (Figure A1d). ImageJ software (NIH, Bethesda, MD, USA) was used to measure the respective areas in 2–4 sections per slide. The volumes of the infarct core, reactive astrogliosis in peri-infarct area and densely packed microglia were calculated for each mouse from respective areas, the number of analyzed sections and the section thickness.

### 4.6. Immunofluorescence and Analysis of Newly Born Cells in the Per-Infarct Area

Antigen retrieval was performed by treatment of free-floating sections with 2 N HCl (10 min, 37 °C) followed by 0.1 M borid acid (10 min). After washing sections were incubated with rat anti-BrdU-antibody (1:500, AbD Serotec, Raleigh, NC, USA, OBT0030CX) and rabbit anti-GFAP (1:1000, Dako Agilent Technologies, Santa Clara, USA, Z0334), rabbit anti-IBA1 (1:500, Wako Industries, Osaka, Japan, 019-19741), the neuron marker NeuN (Merck Milipore, Billerica, MA, USA ABN78) overnight at 4 °C. Following further washing, sections were incubated with a mixture of Alexa-Fluor 488 goat anti-rat (1:500, Life Technologies, NC, USA, A-11006) and Alexa-Fluor 568 goat anti-rabbit (1:500, Life Technologies, A-11036) for 3 h at room temperature and darkness. Slices were washed and mounted on slides. Slides were air-dried and cover-slipped with Mowiol.

Newborn cells were analyzed in a defined region within the peri-infarct area (Figure A1e,f) in male and female *Bmal1*^+/+^ mice (*n* = 3; 7d ♂ *Bmal1*^+/+^ *n* = 3; 7d ♀ *Bmal1*^+/+^ *n* = 3; 14d ♂ *Bmal1*^+/+^ *n* = 3; 14d ♀ *Bmal1*^+/+^ *n* = 3) and *Bmal1*^−/−^ mice (*n* = 3; 7d ♂ *Bmal1*^−/−^ *n* = 3; 7d ♀ *Bmal1*^−/−^ *n* = 3; 14d ♂ *Bmal1*^−/−^ *n* = 3; 14d ♀ *Bmal1*^−/−^ *n* = 3). An observer blind to the experimental condition performed image analysis. Immunofluorescent cells were counted in z-stacks (2 µm) of microphotographs obtained by confocal laser scanning microscopy (LSM 510 Meta, Carl Zeiss, Oberkochen, Germany). The total number of BrdU-positive (BrdU^+^) cells was counted and the number of double-labelled cells for BrdU and GFAP (BrdU^+^/GFAP^+^-cells) as well as BrdU and IBA1 (BrdU^+^/IBA1^+^-cells) were determined.

### 4.7. Analysis of Gonadotropin Releasing Hormone (GnRH) Positive Neurons and Fibers

Female 10–12 weeks old *Bmal1*^+/+^ (*n* = 4) and *Bmal1*^−/−^ (*n* = 4) mice were used. Brains, including hypothalamus, were sectioned coronally (30 µm thickness) by using a cryomicrotome (Reichert-Jung) in 6 parallel series. One of these series was stained with immunohistochemistry as described above using a rabbit polyclonal anti GnRH (1:4000, Immunostar, Hudson, WI, USA). Image acquisition was performed using bright field mode on a Keyence BZ 900E microscope (Keyence, Osaka, Japan) with settings kept constant through image acquisition. An observer blind to the genotype performed image analysis. GnRH-immunoreactive neurons were counted manually in the preoptic area (POA) (0.62–0.38 mm rostral from bregma, according to Franklin and Paxinos mouse brain atlas). GnRH-immunoreactive fibers were analyzed in POA and at their projection to the median eminence (ME) using ImageJ software. Data was expressed as percentage of area covered with GnRH fibers relative to the total area.

### 4.8. Estradiol Assay

For the estradiol assay in female *Bmal1*^+/+^ (*n* = 19) and *Bmal1*^−/−^ mice (*n* = 14), blood was drawn from the right atrium, collected in EDTA sample tubes and centrifuged at 4 °C for 15 min at 1400× *g*. From the supernatant/plasma 25 µL were subjected to estradiol-ELISA (SE-120049, Sigma Aldrich) according to the manufacturer’s protocol. Plates were read at 450 nm in a plate reader (Multiskan FC, ThermoScientific, Waltham, MA, USA). Assay sensitivity ranged from 10 to 1000 pg/mL.

### 4.9. Statistical Analysis

Data was statistically analyzed using GraphPad Prism software (GraphPad Software, Inc., La Jolla, CA, USA). Data are presented as mean. Due to special hypotheses concerning the difference between two groups, respectively, pairwise comparisons with Mann-Whitney-U Test were performed. Values of *p* < 0.05 were considered significantly different.
